# Insights into in vivo follicle formation: a review of in vitro systems

**DOI:** 10.1007/s00418-021-02058-w

**Published:** 2021-11-30

**Authors:** Ren Tanimoto, Kyota Yoshida, Shinya Ikeda, Yayoi Obata

**Affiliations:** Department of Bioscience, Tokyo University of Agriculture, 1-1-1 Sakuragaoka, Setagaya-ku, Tokyo, 156-8502 Japan

**Keywords:** Anti-Müllerian hormone, α-fetoprotein, Estrogen, Oogenesis, Ovarian culture, Primordial follicle formation

## Abstract

In vitro systems capable of reconstituting the process of mouse oogenesis are now being established to help develop further understanding of the mechanisms underlying oocyte/follicle development and differentiation. These systems could also help increase the production of useful livestock or genetically modified animals, and aid in identifying the causes of infertility in humans. Recently, we revealed, using an in vitro system for recapitulating oogenesis, that the activation of the estrogen signaling pathway induces abnormal follicle formation, that blocking estrogen-induced expression of anti-Müllerian hormone is crucial for normal follicle formation, and that the production of α-fetoprotein in fetal liver tissue is involved in normal in vivo follicle formation. In mouse fetuses, follicle formation is not carried out by factors within the ovaries but is instead orchestrated by distal endocrine factors. This review outlines findings from genetics, endocrinology, and in vitro studies regarding the factors that can affect the formation of primordial follicles in mammals.

## Introduction

In mammals, follicle formation begins before or immediately after birth (Baker 1963; Rüsse 1983; Pepling 2012). When oogonia that differentiate from primordial germ cells (PGCs) in fetal ovaries complete their final DNA replication, they transition to the meiotic phase; the oocytes are then differentiated. The transition from oogonia to oocytes proceeds in cysts. Then, primordial follicles form over a series of events: oocyte cyst breakdown, oocyte death, and the encapsulation of oocytes by pre-granulosa cells. In addition, pre-granulosa cells stop dividing even before primordial follicle formation. In female mammals, most primordial follicles that form around birth become dormant and have lifelong reproductive resources. Afterwards, a cohort of dormant primordial follicles in the ovaries is activated to transition to the growth stage. This process of transitioning to the growth stage generally consists of a first wave followed by subsequent waves; differences in the primordial follicles activated in the first and subsequent waves have been gradually uncovered (Ford et al. 2020). When primordial follicles transition to the growth stage, the flat pre-granulosa cells become cuboidal and resume mitosis. In the process of growing from primary follicles covered with a layer of granulosa cells into secondary follicles covered with two or more layers of granulosa cells, theca cells differentiate in the ovaries (Richards et al. 2018). Oocytes, granulosa cell layers, a basement membrane, and theca cell layers are arranged regularly to create the follicular structure. As a follicle grows, a follicular antrum forms, and the granulosa cells adjacent to the oocytes differentiate into cumulus cells. These cells have properties different from those of the mural granulosa cells that are distal to the oocytes (along the theca cell layer of the follicle). In monovulatory species, a dominant follicle is selected during follicular growth, following which some of them regress (Ginther 2021). Finally, an intrinsic number of oocytes ovulate in the unique estrous cycles of these species.

Recently, ovarian culture systems have been established to reproduce oocyte/follicle formation in vitro from mouse fetal ovaries containing PGCs, and from reconstituted ovaries with PGC-like and fetal ovarian somatic cell-like cells, both of which are differentiated from pluripotent stem cells (Hikabe et al. 2016; Morohaku et al. 2016b; Morohaku 2019; Yoshino et al. 2021). Current in vitro systems do not fully reproduce in vivo oogenesis including folliculogenesis (Nagamatsu et al. 2019; Shimamoto et al. 2019; Nagamatsu 2021; Tanimoto et al. 2021). Elucidating the causes of abnormal oogenesis in vitro will lead to a better understanding of the processes of normal oocyte and follicle formation in vivo and the renovation of in vitro systems. Furthermore, to understand the etiology of human reproductive diseases, such as polycystic ovary syndrome and premature ovarian failure, it is important to gain an integrated understanding of the programs of oocyte, granulosa cell, and theca cell differentiation, and the endocrine environment during follicle formation and growth. Our recent in vitro study suggest that a factor secreted from mouse fetal liver is indispensable for ovarian follicle formation (Tanimoto et al. 2021). To the best of our knowledge, the *trans*-acting factors that are essential for normal follicle assembly are thus far unknown. This review outlines findings from genetics, endocrinology, and in vitro studies on factors that can affect follicle formation; it mainly focuses on mice, but follicular formation processes in other mammals are also addressed.

### Events occurring during primordial follicle assembly

In the embryonic stage, oogonia/oocytes are connected by intracellular bridges and maintain a structure called a germline cyst or oocyte cyst. The biological significance of this oocyte cyst structure has drawn attention because it is preserved in various animal species, including *Drosophila* (Spradling 1993), *Xenopus* (Kloc et al. 2004), and humans (Gondos 1973). Organelles such as mitochondria, endoplasmic reticulum, and Golgi vesicles move into oogonia/oocytes in the cyst via intracellular bridges. In mice, the donor oocytes of the organelle die, and the recipient oocytes that receive the organelles survive to become primary oocytes in the primordial follicle. However, it is not clear which oocytes are destined to die, or whether this process is selective or random. In contrast, in *Drosophila*, only one oogonium out of a cyst consisting of 16 sister oogonia differentiates into an oocyte; the remaining oogonia differentiate into nurse cells to foster oocytes. A structure called the fusome causes asymmetric organelle distribution between sister oogonia, which determines which oogonium differentiates into an oocyte (Huynh and St Johnston 2004). In any case, this cyst structure is believed to be a female reproductive strategy for producing high-quality oocytes in the ovary.

Testis-expressed gene 14 (*Tex14*) is a gene that is involved in intracellular bridge formation. In *Tex14*-null female mouse fetuses, oocytes have been shown to lack stable intracellular bridges, but the oocyte cyst structure does not disappear completely. Although it remains unclear whether cysts are essential for oogenesis in mammals, primordial follicles have been shown to form in *Tex14*-null mice (Ikami et al. 2021). In addition, *Tex14*-null mice will remain fertile for over a year. These findings indicate that normal intracellular bridge structures are not necessary for primordial follicle formation (Greenbaum et al. 2009).

Apoptosis occurs in mouse oocytes during oocyte cyst breakdown. Deleting the caspase 2 or BCL2-associated X protein (*Bax*) gene, which induces apoptosis, increases the number of oocytes and primordial follicles by suppressing apoptosis (Bergeron et al. 1998; Perez et al. 2007). *Bax*-knockout mice have more PGCs and oocytes than wild-type mice, but more of their oocytes also die from apoptosis during primordial follicle formation (Greenfeld et al. 2007). Therefore, while apoptosis controls the number of follicles, it is unclear whether it is essential for the formation of normal primordial follicles.

Multi-oocyte follicles (MOFs) are associated with abnormal oocyte cyst breakdown, regardless of the cause (Pepling 2012). As the oocytes in an MOF are individually separated, and are no longer connected by intracellular bridges, abnormal oocyte cyst breakdown represents a delay, rather than a complete inhibition. MOFs may be caused by a time lag between cyst breakdown and the surrounding of oocytes by pre-granulosa cells. Oocytes collected from MOFs show lower fertilization rates than those from normal follicles, and female mice exhibiting MOFs have reduced fertility (Iguchi et al. 1991; Vanorny et al. 2014). Therefore, a normal follicle’s structure is important for ensuring female fertility. Several factors have been reported to cause MOFs. Here, *Notch2*, *Jag1/Jagged 1*, activin and inhibin, and estrogen are discussed.

### Function of Notch signaling during primordial follicle formation

Notch signaling is an essential factor in determining the fate of cell differentiation during ontogeny; it is widely evolutionarily preserved. Notch is a single transmembrane receptor that is encoded by *Notch1*, *Notch2*, *Notch3*, and *Notch4* in mice. Moreover, Notch ligands are encoded by *Jag1*, *Jag2*, delta-like canonical Notch ligand 1 (*Dll1*), *Dll3*, and *Dll4* in mice (Vanorny and Mayo 2017). The glycosylation of the Notch extracellular domain is essential for binding to ligands and activating Notch signaling. Furthermore, lunatic fringe (LFNG) plays a role in glycosylation during the formation of follicles (Hahn et al. 2005). When Notch binds to a ligand, a disintegrin and metallopeptidase domain 10 (ADAM10) cleaves the extracellular domain from the cell membrane (Feng et al. 2016). The intracellular domain cleaved by γ-reductase then enters the nucleus and activates the transcription of the hairy/enhancer-of-split (*hes*) and hes-related with YRPW motif (*Hey*) family genes (Trombly et al. 2009). These genes also encode transcription factors, but it is not clear which genes they directly bind to when activating transcription during follicle formation. The functions of Notch2 and JAG1 in the formation of primordial follicles have been previously reported. Notch2 is expressed in pre-granulosa and granulosa cells, whereas JAG1 is expressed in oocytes. Significantly higher MOF formation has been observed following the deletion of *Notch2* from pre-granulosa cells, and following the deletion of *Jag1* from oocytes. Deleting *Notch2* from pre-granulosa cells or inhibiting Notch signals with an inhibitor in a culture of neonatal mouse ovaries have both been shown to significantly increase the number of oocytes remaining in a cyst. The number of primordial follicles has been shown to decline significantly under these conditions (Trombly et al. 2009; Xu and Gridley 2013; Vanorny et al. 2014).

### Regulation of primordial follicle formation by activin and inhibin

Activin belongs to the transforming growth factor-beta (TGF-β) superfamily. Activin consists of an inhibin βA subunit and/or a βB subunit. Homodimers of βA subunits are termed activin A, homodimers of βB subunits are termed activin B, and heterodimers of the βA and βB subunits are termed activin AB (Mason et al. 1985; Forage et al. 1986; Mayo et al. 1986). Activin has been isolated from porcine follicular fluid as a substance that promotes the secretion of follicle-stimulating hormone (FSH) (Ling et al. 1986; Vale et al. 1986). Prior to the discovery of activin, inhibin was proposed to be a factor in controlling gonadotropin levels. Inhibin consists of an inhibin α subunit and a βA or βB subunit, the former being inhibin A and the latter being inhibin B (Mason et al. 1985; Forage et al. 1986; Mayo et al. 1986). The inhibin α, βA, and βB subunits are encoded by *Inha*, *Inhba*, and *Inhbb*, respectively. These glycoproteins are secreted from pre-granulosa and granulosa cells that have no physiological activity until their N-terminal sides are cleaved. Inhibin possesses an anti-activin action. As a model, it is thought to inhibit the functions of activin by competing for subunit assembly or receptor binding (Makanji et al. 2014). Furthermore, follistatin is known to bind to activin and inhibit its action. When activin binds to dimeric type II receptors (ActRII or ActRIIB), they recruit dimeric type I receptors (ACVR1B/ALK4) that are phosphorylated by the serine/threonine kinase activity of type II receptors. Subsequently, small mothers against decapentaplegic (Smad)2 and Smad3 are phosphorylated; they then bind to Smad4 (Makanji et al. 2014). This trimer translocates to the nucleus to activate transcription. Mice in which *Inhba* has been deleted cannot produce activin A, activin AB, or inhibin A. *Inhba*-null mice have been observed to die after birth, although the relevant report did not mention the effects of this treatment on follicle formation (Matzuk et al. 1995). If *Inhbb* is deleted, activin B, activin AB, and inhibin B cannot be produced. *Inhbb*-null mice are viable, and males are fertile, although most offspring from *inhbb*-null females die close to birth. This phenomenon has not been attributed to ovarian abnormalities because it has been shown that there is no difference in the number of concepti compared with those in wild-type females (Vassalli et al. 1994). However, within in vitro systems, activin has been shown to promote the proliferation of oogonia in human fetal ovaries (Martins da Silva et al. 2004). Furthermore, the in vitro administration of mouse activin before follicle formation has been shown to greatly increase the number of primordial follicles (Bristol-Gould et al. 2006). Meanwhile, it has been established that *Inha*-null mice cannot produce inhibin A or inhibin B. *Inha*-null mice can be born, but they are infertile due to tumors that develop in their gonads (Matzuk et al. 1992), although no abnormalities have been observed regarding primordial follicle formation or MOFs (Myers et al. 2009). Follistatin is encoded by *Fst*, which produces three variants: FST288, FST303, and FST315. In the ovaries of mice in which both FST303 and FST315 have been deleted (that is, mice that can only express FST288), oocyte cyst breakdown has been shown to be delayed compared with wild-type mice. In addition, the frequency of oocyte apoptosis during follicle formation has been found to decline in the ovaries of these mice (Kimura et al. 2011). Furthermore, when FST288 was incorporated into mouse fetal ovaries in vitro, many oocytes were found to remain in the cyst, and the proliferation of granulosa cells was shown to be suppressed (Wang et al. 2015). These findings indicate that activin promotes primordial follicle formation, and that follistatin appears to have an inhibitory effect on primordial follicle formation by delaying oocyte cyst breakdown.

### Effects of estrogen on primordial follicle formation

The effects of estrogen on primordial follicle formation vary between different animal species. In baboons, which are nonhuman primates, a small number of primordial follicles have been shown to appear in fetal ovaries on day 100 of gestation (gestation period 184 days). In baboons, estrogen is synthesized in the placenta, and estradiol (E2) levels in maternal blood rise from day 85 to day 165 during gestation (Zachos et al. 2002). If the aromatase inhibitor letrozole is administered to the mother during this period, maternal E2 levels decline. When an aromatase inhibitor was administered, more oogonia/oocytes were observed in nests with significantly fewer follicles. The nest is an oogonia/oocyte cyst in the ovigerous cord. In addition, baboons that were administered an aromatase inhibitor exhibited increased blood levels of testosterone and androstenedione, indicating that this inhibitor specifically inhibits the conversion of these hormones to estrogen. The simultaneous administration of an aromatase inhibitor and E2 has been shown to lead to the recovery of the ovarian phenotype. These findings indicate that, among the steroid hormones, it is not androgen, but estrogen, that affects follicle formation (Zachos et al. 2002). Although the cell clusters, termed as “interfollicular nests” in this report, appear to differ from mouse oocyte cysts, it is clear that follicle formation in baboons is promoted by estrogen.

In cows, primordial follicles have been observed to appear in fetal ovaries on day 90 of gestation (term pregnancy 280 days). The number of primordial follicles in the fetal ovaries was subsequently found to increase, peaking during days 140–210 of gestation (Yang and Fortune 2008). Cows and baboons exhibit significantly different maternal blood E2 levels during pregnancy, with levels in cows being nearly 100 times lower than those in baboons. Yang and Fortune (2008) cultured bovine fetal ovaries at various stages of pregnancy and measured the concentration of E2 secreted in the medium to determine the E2 levels produced locally during follicle formation. They found that levels gradually decreased after peaking on days 80–100, were undetectable on days 141–193, and then began to rise again from day 210 (Yang and Fortune 2008). This indicates that E2 secretion is repressed during primordial follicle formation. Cytochrome P450, family 19, subfamily a, polypeptide 1 (CYP19A1)/aromatase has been found to be expressed in rete and stromal cells on day 130, but is expressed thereafter in granulosa cells of growing follicles other than primordial follicles. The in vitro culture of ovarian pieces from fetuses on days 90–140 with a medium supplemented with E2 did not increase the number of primordial and primary follicles (Yang and Fortune 2008). Thus, estrogen exerts an inhibitory action on the formation of bovine primordial follicles and their activation.

In contrast, many studies have examined how exogenous estrogen inhibits the progression of oocyte cyst breakdown in mice, resulting in MOFs. The factors that bind to estrogen receptors include endogenous E2, estriol (E3), estrone (E1), the synthetic estrogen diethylstilbestrol (DES), the plant-derived natural compounds genistein and coumestrol, and endocrine disruptors such as bisphenol A. In mice, oocyte cysts in fetal ovaries begin to break down on day 17.5 of gestation; primordial follicles are formed by approximately postnatal day 7 (day of birth = day 1) (Pepling 2012). Administering E2 (5 mg kg^−1^ day^−1^) to mice from 1 to 4 days after birth has been found to halve the number of primordial follicles in the ovaries, while doubling the number of oocytes that remain in cysts (Chen et al. 2007). MOFs have been frequently observed in ovaries approximately 2 weeks after E2 administration. When DES (1 µg day^−1^) was administered to mice from 1 to 5 days after birth, MOFs containing 2–23 oocytes each were observed 1 month later (Iguchi et al. 1990). Similarly, when genistein (50 mg kg^−1^ day^−1^) was administered to mice from 1 to 5 days after birth, the number of primordial follicles decreased and the number of oocytes remaining in cysts increased (Jefferson et al. 2006). Focusing on endogenous E2 production, the expressions of 3β-hydroxysteroid dehydrogenase (HSD3B) and CYP19A1/aromatase suggest that E2 production occurs in the fetal placenta (Blomquist et al. 1993; Raunig et al. 2011), ovaries (Dutta et al. 2014), and brain (McCarthy 2008). In mice, the concentration of E2 in fetal blood is higher than that in maternal blood during pregnancy; it decreases rapidly from day 17.5, when oocyte cysts begin to break down, and remains low until day 7 after birth (Dutta et al. 2014). This decline in the concentration of E2, which can inhibit follicle formation, seems to trigger the breakdown of oocyte cysts.

### *Insights into in vivo follicle formation from in vitro investigations into causes of abnormal follicle formation*

**Fig. 1:**
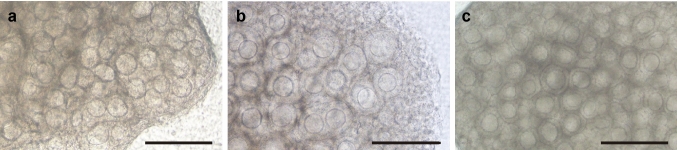
Failure in secondary follicle formation in in vitro-cultured mouse fetal ovary. **a** Fetus-derived ovary on day 17 of culture. Ovaries were cultured with a conventional medium containing fetal bovine serum (FBS). This ovary contains numerous growing oocytes, but they have not formed a clearly visible follicle structure. **b** Newborn-derived ovary on day 10 under the same culture conditions. Growing oocytes are enclosed within secondary follicles. **c** Fetus-derived ovary on day 17 of culture with a medium containing FBS and estrogen receptor antagonist ICI 182,780. Hypoplastic follicles were recovered by inhibiting estrogen receptors. Bars, 200 µm

**Fig. 2:**
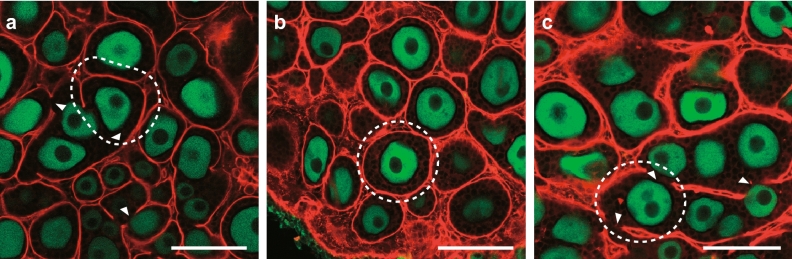
Abnormal arrangement of laminin basement membrane in in vitro-cultured mouse fetal ovary. Follicular basement membrane is labeled in red and oocytes in green using anti-Laminin and anti-DDX4 antibodies, respectively. The areas surrounded by white dashed lines are examples of a follicle area that should be enclosed with laminin. Arrow heads indicate absence of laminin arrangement. **a** Fetus-derived ovary cultured with a conventional medium containing FBS on day 17 of culture. The wrapping an individual follicle by laminin envelope is interrupted. **b** Fetus-derived ovary cultured with a medium containing SPS instead of FBS on day 17 of culture. If FBS that possibly provides estrogen and/or estrogen-like substances is excluded in culture during primordial follicle assembly, laminin envelope completely covers single follicle. **c** Fetus-derived ovary cultured with medium supplemented with exogenous AMH under the same conditions as in **b** on day 17 of culture. AMH is ectopically expressed by activation of estrogen signaling. Even if FBS-free medium is used, laminin envelope again fails in enclosing individual follicles in the cultured ovary by AMH addition to the medium. Bars, 100 µm

We have established an in vitro system that is capable of achieving the entire process of oogenesis (Morohaku et al. 2016b; Morohaku 2019). The main focus of this study has been the production of fertile oocytes from the gonads of mouse fetuses containing only mitotic PGC/oogonia. An in vitro system that cannot produce fertile oocytes thus cannot reproduce the spontaneously occurring in vivo process of oogenesis. An in vitro system that can reproduce the process of oogenesis would not only serve as a tool for understanding the mechanisms of oocyte/follicle development and differentiation, but could also help increase the production of useful livestock and genetically modified animals, and identify the causes of infertility in humans. Initial attempts have been made to produce secondary follicles by assembling and growing, then using organ cultures of mouse fetal gonads at 12.5 days of gestation. On culture day 17, it was expected that secondary follicles equivalent to those in the ovaries of in vivo 10-day-old mice would be produced. However, despite observing a large number of growth-stage oocytes in the cultured ovaries, almost no obvious follicle structures were found (Fig. 1a) (Morohaku et al. 2016b; Morohaku 2019). Furthermore, attempts to retrieve secondary follicles from the ovaries only recovered a few follicles per ovary; those follicles that were isolated were MOFs. Immunofluorescent staining for laminin, which constitutes the basement membrane, showed that these follicles were not encapsulated in basement membranes, resulting in follicular dysplasia (Fig. 2a) (Morohaku et al. 2016b; Tanimoto et al. 2021). In contrast, when the ovaries of 0-day-old newborn mice were cultured for 10 days, secondary follicles were formed (Fig. 1b) (Eppig and O'Brien 1996; Morohaku et al. 2016a).

To investigate the underlying causes, RNA sequencing (RNA-seq) analysis was performed on ovaries from newborn mice, and on ovaries collected from mouse fetuses at 12.5 days of gestation that had been cultured for 7 days, to compare transcripts (Morohaku et al. 2016b). Differentially expressed genes (DEGs) were extracted, and upstream regulators were analyzed. Putative factors such as E2, SP1, and CTNNB1/beta-catenin were identified. As described above, estrogen inhibits normal follicle formation in mice. The liganded-estrogen receptors ESR1/ERα and ESR2/ERβ bind to palindromic estrogen-responsive elements (ERE; 5′-GGTCANNNTGACC-3′) and regulate their transcription. Estrogen receptors function as homodimers or heterodimers (Kuiper et al. 1997). For example, ESR1 and Sp1 heterodimerize, ESR1 binds to ERE half sites, and Sp1 binds to nearby Sp1 binding sites to regulate transcription (Su and Song 2016). In contrast, estrogen binds to the G-protein-coupled receptor GPER1/GPR30 on cell membranes, and has also been shown to contribute to signal transduction, such as the phosphorylation of Erk1/2 (Filardo et al. 2000). RNA-seq analysis results and other reports have suggested that excessive estrogen signaling may change the gene expression profiles of in vitro-cultured ovaries, resulting in abnormal follicle formation. However, in vitro systems have been isolated from the circulation of estrogen derived from the placenta and maternal blood. Therefore, the possible factors affecting estrogen signaling include (1) the overexpression of estrogen receptors, (2) excess estrogen production by the ovaries in in vitro environments, and/or (3) medium containing estrogen. Various analyses have shown that the addition of fetal bovine serum (FBS) to medium can be an issue in this regard (Morohaku et al. 2017). The addition of a serum protein substitute (SPS) instead of FBS or the addition of estrogen receptor antagonist ICI 182,780 (ICI) and FBS to the medium during culturing days 5–11 (equivalent to 17.5 days of gestation to 4 days after birth, in which follicle assembly occurs) can both dramatically improve follicle formation. They have also been shown to greatly increase the number of secondary follicles collected from individual ovaries on day 17 of culture (Fig. 1c). Furthermore, the follicular basement membranes were found to be clearly surrounded by laminin, and single oocytes were shown to be enclosed within a single follicle on day 17 of culture (Fig. 2b) (Morohaku et al. 2016b). Focusing on the earlier development stage, on culture days 7–13, oocyte cyst breakdown was shown to be delayed in ovaries that were cultured in a conventional medium containing FBS, compared with ovaries cultured in a medium containing FBS and ICI (Tanimoto et al. 2021). This indicates that abnormal follicle formation occurring in the in vitro culturing of mouse fetal ovary is mediated by estrogen receptors, and that it is accompanied by delayed oocyte cyst breakdown.

**Fig. 3:**
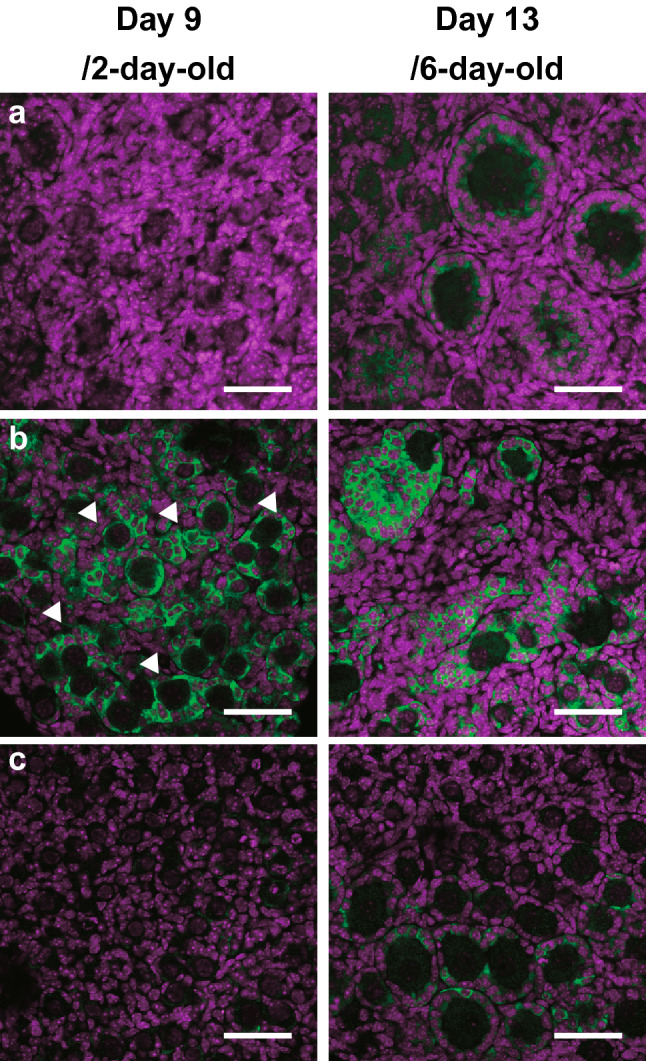
Premature expression of AMH in in vitro-cultured mouse fetal ovary. **a** AMH expression in in vivo-derived ovaries. AMH is not expressed in an ovary from 2-day-old mouse (left), but detected in granulosa cells in primary or greater follicles in an ovary from 6-day-old mouse (right). **b** AMH expression in an ovary cultured with a conventional medium containing FBS. AMH is prematurely expressed in an ovary on day 9, which corresponds to 2-day-old mouse in vivo (left). Arrow heads indicate premature AMH expression in ovarian somatic cells. **c** AMH expression in an ovary cultured with a medium containing FBS and ICI. Repression of the ectopic expression of AMH in an ovary on day 9 is achieved by blocking of estrogen signaling (left). In contrast, AMH expression is prominent in primary follicles that are formed in an ovary on day 13 (right). AMH is labeled in green and nuclei in magenta using DAPI. Reuse and modify the images published in *Development* (Tanimoto et al. 2021). Bars, 50 µm

The candidate genes that cause abnormal follicle formation have also been explored (Table 1) (Morohaku et al. 2016b; Tanimoto et al. 2021). The DEGs of newborn mouse ovaries and cultured ovaries were found to include anti-Müllerian hormone (*Amh*), *Inha*, and *Lfng*. The extreme overexpression of *Amh* has been observed in cultured ovaries, compared with that in newborn mouse ovaries (Tanimoto et al. 2021). *Amh* causes the regression of the Müllerian ducts, which in male fetuses are secreted from Sertoli cells and differentiate into oviducts and other female reproductive tracts. Both the testes and female genitalia have been shown to differentiate in XY mice lacking *Amh* (Behringer et al. 1994). In females, it has been suggested that *Amh* is expressed in granulosa cells in growth-stage follicles that start in primary follicles, and that it has an inhibitory function against the activation of primordial follicles (Durlinger et al. 1999). Immunostaining analysis of AMH has revealed it to be expressed in granulosa cells in primary or later follicles emerging approximately from day 6 after birth in vivo (Fig. 3a), while in cultured ovaries AMH expression has been observed early (at the equivalent of age 0 day in ovarian somatic cells, presumably granulosa cells, Fig. 3b) (Tanimoto et al. 2021). The premature expression of AMH has also been abolished in cultured ovaries by adding ICI (Fig. 3c). Furthermore, when AMH was added to a medium in which FBS was replaced with SPS, the recovery rate of the secondary follicles was found to decline in a concentration-dependent manner. In addition, the surrounding of single oocytes by granulosa cells and basement membranes disappeared (Fig. 2c) (Tanimoto et al. 2021). These findings are consistent with reports that the incorporation of AMH inhibits the in vitro formation of primordial follicles in newborn rat ovaries (Nilsson et al. 2011). AMH is a member of the TGF-β superfamily. AMH signaling is essential for the type II receptor AMHR2 and the type I receptor BMPR1A/ALK3, which control cell fate via the phosphorylation of Smad1 and Smad5 (Mishina et al. 1996; Jamin et al. 2002). The addition of AMH to primary cultures of granulosa cells collected from the ovaries of 3-week-old mice has been found to increase the expression of inhibitor of DNA binding 3 (*Id3*) (Sèdes et al. 2013), although it was unclear whether this effect was direct or indirect; in ovaries cultured in our study, the expression of *Id3* did not increase (Table 1). To date, the mechanism through which AMH inhibits follicle formation has remained unclear. However, the increase in the expression of forkhead box L2 (*Foxl2*) in cultured ovaries suggests that pre-granulosa cells differentiate into granulosa cells before follicle assembly. Furthermore, the overexpression of *Inha* and the silencing of *Lfng* in cultured ovaries (Table 1) are consistent with abnormal primordial follicle formation as observed in the ovaries of *Inha* transgenic and *Lfng*-null mice (McMullen et al. 2001; Hahn et al. 2005). As there are no reports of the TGFβ superfamily of inhibin, activin, and AMH competing for the receptors, there is currently no evidence that AMH participates with inhibin to inhibit follicle formation.

**Table 1:** Expression of key genes associated with primordial follicle formation in the neonatal ovaries and fetal-derived ovaries on day 7 of culture

	Gene symbol	NSV			Note
		In vitro (FBS)	In vitro (ICI)	In vivo	
Intracellular bridge	*Tex14*	12.07	12.06	11.62	Stabilization of midbody
	*Kif23/Mklp1*	10.64	10.60	10.99	Promotion of cytokinesis
	*Cep55*	10.24	10.27	10.26	Promotion of cytokinesis
	*Racgap1*	11.99	11.95	12.58	Promotion of cytokinesis
	*Pdcd6ip/Alix*	13.89	13.84	13.73	Promotion of cytokinesis
	*Tsg101*	11.89	11.88	12.02	Promotion of cytokinesis
Apoptosis	*Bax*	12.62	12.67	12.70	Promotion of apoptosis
	*Casp2*	12.79	12.74	12.92	Promotion of apoptosis
	*Casp3*	11.57	11.61	11.28	Promotion of apoptosis
	*Smpd1*	12.07	12.09	11.14	Promotion of apoptosis
	*Mcl1*	13.60	13.61	13.53	Inhibition of apoptosis
	*Bcl2*	12.04	11.67	11.09	Inhibition of apoptosis
Notch signaling	*Notch1*	11.31	11.14	10.78	Receptor
	*Notch2*	11.97	11.98	12.73	Receptor
	*Notch3*	11.02	10.64	10.67	Receptor
	*Notch4*	9.84	9.17	8.48	Receptor
	*Jag1/Jagged1*	11.58	11.42	11.28	Ligand
	*Jag2/Jagged2*	13.78	13.87	14.28	Ligand
	*Dll1*	8.92	8.76	7.83	Ligand
	*Dll3*	6.95	7.07	6.51	Ligand
	*Dll4*	10.17	9.27	8.41	Ligand
	*Lfng*	6.80	6.81	8.64	Glycosylation
	*Psen1*	13.03	12.96	12.60	γ-reductase subunit
	*Psen2*	11.92	11.94	11.59	γ-reductase subunit
	*Ncstn*	13.41	13.31	13.24	γ-reductase subunit
	*Psenen*	8.40	8.51	8.53	γ-reductase subunit
	*Aph1a*	9.75	9.59	9.44	γ-reductase subunit
	*Aph1b*	10.61	10.65	10.20	γ-reductase subunit
	*Adam10*	13.73	13.84	13.63	Metallopeptidase
	*Hes1*	12.26	12.19	12.05	Notch target
	*Hes2*	0.01	0.38	2.36	Notch target
	*Hes3*	3.02	3.32	3.21	Notch target
	*Hes5*	3.74	3.13	4.06	Notch target
	*Hes6*	11.30	11.45	11.66	Notch target
	*Hes7*	4.26	4.95	4.75	Notch target
	*Hey1*	11.45	11.36	10.53	Notch target
	*Hey2*	10.63	10.09	9.20	Notch target
Activin	*Inha*	14.34	13.82	12.68	Glycoprotein
	*Inhba*	4.61	5.07	5.37	Glycoprotein
	*Inhbb*	12.43	12.24	11.64	Glycoprotein
	*Fst*	11.51	11.71	12.37	Glycoprotein, inhibitor of activin
	*Acvr2a/ActRII*	10.95	11.05	11.25	Type II receptor for activation and inhibin, etc
	*Acvr2b/ActRIIB*	8.95	9.00	9.02	Type II receptor for activation and inhibin, etc.
	*Acvr1b/Alk4*	11.12	11.11	11.00	Type I receptor for activation, etc.
	*Acvr1c/Alk7*	4.65	4.84	4.00	Type I receptor for activation, etc
	*Tgfbr3/Betaglycan*	11.24	11.16	11.05	Type III receptor for inhibin
Estrogen	*Esr1*	11.16	11.22	11.79	Receptor for estrogen
	*Esr2*	10.50	10.67	9.93	Receptor for estrogen
	*Gper1*	4.08	4.45	5.91	Receptor for estrogen
	*Sp1*	12.92	12.91	12.75	Co-factor of ESR
	*Hsd3b1*	12.51	13.01	13.02	Estrogen production
	*Cyp19a1/aromatase*	4.42	5.02	5.97	Estrogen production
	*Amhr2*	14.20	14.21	13.57	Type II receptor for AMH
	*Bmpr1a*	13.49	13.65	13.47	Type I receptor for AMH
	*Amh*	11.27	9.84	7.76	Ligand
	*Id3*	12.56	12.89	12.87	Downstream of AMH
Granulosa cell differentiation	*Foxl2*	14.52	14.50	13.91	Transcription factor
	*Lgr5*	10.99	11.03	11.44	Receptor for RSPO1
	*Rspo1*	11.95	12.22	12.88	Ligand
Chemokine	*S100a8*	4.82	3.53	5.36	
Rearrangement of extracellular matrix	*Plau*	10.89	9.82	10.54	
	*Mt1*	10.88	11.59	11.95	
	*Mmp2*	13.38	13.20	14.26	
	*Timp3*	13.41	13.40	12.87	
Serum protein	*Shbg*	8.32	7.62	7.21	
	*Afp*	2.20	2.51	2.29	
	*Alb*	0.06	0.97	2.12	

*NSV* normalized signal value, all of the mRNA expression data were normalized by log2 transformation

Moreover, abnormal follicle formation in the ovaries cultured by a previously developed system has been found to occur through ESR1, among the estrogen receptors (Tanimoto et al. 2021). The inhibition of ESR1 has been shown to lead to recovery from *Amh* overexpression, and to improve secondary follicle isolation. The question of whether ESR1 binds directly to *Amh* has also been investigated; chromatin immunoprecipitation–polymerase chain reaction analysis has revealed that ESR1 binds to the ERE half site of the *Amh* transcription start site at ≥ 4.1 times higher concentrations in cultured ovaries than in newborn ovaries. Follicles are formed and *Amh* is expressed in primary or later-stage follicles, even in the ovaries of female mice lacking *Cyp19a1* (Britt et al. 2004), that is, mice unable to synthesize estrogen. Thus, the transcriptional activation of *Amh* via ESR1 is a phenomenon that occurs in in vitro-cultured fetal ovaries. Therefore, the expression of *Amh* in growth-stage follicles after birth must occur due to a different mechanism. It is also unclear how estrogen mediates the delay in oocyte cyst breakdown in cultured ovaries. Although *Esr1* is transcribed in its full-length form in the ovaries of newborn mice (Tanimoto et al. 2021), when only non-growth-stage oocytes were isolated from ovaries to examine the expression of *Esr1*, a short variant lacking the 5′ end was generally found (Pan et al. 2005; Veselovska et al. 2015; our unpublished data). This suggests that canonical estrogen signals may not be activated in oocytes during follicle formation.

The main question here regards the amount of estrogen contained in FBS. Measurements of estrogen levels in FBS have delivered a value of only 8.5 pg ml^−1^, which is much lower than that of E2 in mouse fetal serum (Dutta et al. 2014; Tanimoto et al. 2021). While FBS might contain other unknown estrogens that bind to estrogen receptors in addition to E2, the affinity of E2 for ESR1 and ESR2 is much higher than those of most other estrogens (Kuiper et al. 1997). It is assumed that fetal ovaries are susceptible to E2 contained in FBS, although physiological in vivo E2 levels do not lead to ectopic *Amh* expression and abnormal follicle formation. Therefore, there may be endogenous antiestrogenic factors in vivo, similar to ICI. Albumin, α-fetoprotein (AFP), and sex hormone-binding globulin (SHBG) are serum proteins that are known to bind to steroid hormones (Hammond 2016). Estrogen does not have a biological function when bound to these proteins, leaving free estrogen to passively diffuse and access target cells. AFP is the major serum protein in fetuses; it is produced in the fetal liver and yolk sac. After birth, AFP is replaced by albumin in serum proteins and decreases dramatically over time in adulthood. In both mice and humans, AFP and albumin are located near the same chromosome, and are thought to be derived from a common ancestral gene (Ingram et al. 1981; Urano et al. 1984). Albumin has a low affinity for steroid hormones, but is present in large concentrations in the blood. SHBG is produced in fetal and adult livers. Compared with albumin, SHBG has 1000 times higher affinity and specificity than androgens and estrogens, but exists at 1000 times lower levels in the serum. Human AFP inhibits the growth of estrogen-dependent breast cancer and uterine cells after birth (Bennett et al. 1998; Mizejewski 2011), but its E2 binding ability is much lower than that observed in rodents (Nishi et al. 1991). As mice that lack AFP accomplish full-term development, AFP does not appear to be essential for ontogeny. However, AFP-null female mice exhibit infertility, with anovulation and uterine abnormalities (Gabant et al. 2002). These phenomena are related to the sexual differentiation of the brain. The expression of *Cyp19a1* in the brains of male mouse fetuses is much higher than that in female fetuses. Furthermore, large amounts of E2 would be produced locally from testosterone in male fetuses. It is believed that E2 circulating in fetal blood loses its function in the brain because if it binding to AFP. As a result, the brains of AFP-null female fetuses become exposed to free E2 and thus are masculinized. This causes dysfunction regarding gonadotropin-releasing hormone neurons and thus leads to infertility in females (Bakker et al. 2006). Therefore, AFP may play a role in the fact that physiological levels of E2 do not inhibit follicle formation in mouse fetuses. In cultures of mouse fetal ovaries in which AFP was added to a conventional medium containing FBS, follicle formation was not inhibited, while the ectopic expression of *Amh* was suppressed (Tanimoto et al. 2021). This suggests that AFP may regulate the action of E2 during in vivo follicle formation. In fact, blood AFP levels in newborn mice have been found to be much higher than E2 levels (2.5 mg ml^−1^ versus ≤ 5 pg ml^−1^) (Tanimoto et al. 2021). This is consistent with the fact that, despite DES having a greater affinity for estrogen receptors than E2, MOFs are induced by the administration of large doses of DES (Iguchi et al. 1990). In mice, AFP is the most likely candidate for assuring normal follicle formation under circulating physiological levels of E2. Although the binding affinity of bovine AFP and E2 is not known, AFP levels have been shown to peak at 4 months of gestation (6.1 mg ml^−1^) (Abe et al. 1976). Bovine AFP may also affect follicle formation by modulating E2 action in vivo.

Is the regulation of estrogen action a common mechanism used in mammals to achieve successful follicle assembly? In baboons, the administration of aromatase inhibitors to pregnant mothers significantly reduces estrogen levels in maternal blood and inhibits follicle formation, while inhibin levels rise in fetal ovaries (Zachos et al. 2002; Billiar et al. 2003). Increases in *Inha* have also been observed in cultured and mouse ovaries administered with DES (Oikawa et al. 2019; Tanimoto et al. 2021). Changes in estrogen levels may affect the expression levels of common downstream factors that induce abnormal follicle formation. Among mammals, estrogen has differing effects on follicle formation; however, it is important to appropriately regulate its action.

### *Unresolved problems regarding in vitro follicle formation*

**Fig. 4:**
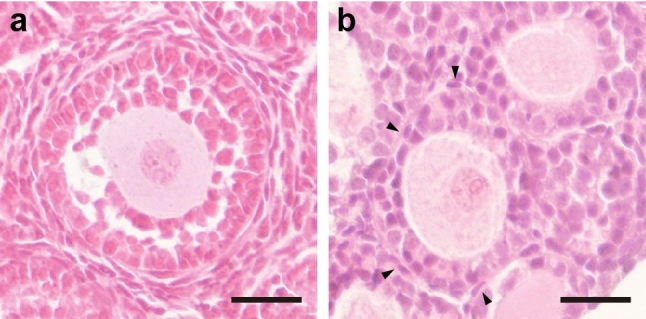
Follicles formed in a cultured ovary by blocking estrogen receptors. Ovarian sections of 10-day-old mice (**a**) and fetus-derived ovaries cultured in a medium containing FBS and ICI on day 17 (**b**). This follicle is enclosed with only a few theca cells (arrow heads). Bars, 25 µm

Estrogens such as genistein and DES form MOFs through ESR2 (Jefferson et al. 2002; Kirigaya et al. 2009). MOFs have been shown to form when genistein or DES is administered to ESR1 null mice, while the administration of genistein or DES to ESR2 null mice has been shown not to create MOFs. Abnormal follicle formation in cultured ovaries has mainly been found to occur via ESR1 (Tanimoto et al. 2021), but ESR1/ESR2-null mouse fetal ovaries need to be examined to ascertain the involvement of ESR2. Moreover, it is difficult to conclude that abnormal follicle formation in cultured ovaries is caused only by estrogen in FBS. For example, MOFs are phenotypes that are found not only following exposure to estrogen but also following *Notch2*, *Jag1*, or *Lfng* deficiencies, or following the overexpression of *Inha* (Iguchi et al. 1990; McMullen et al. 2001; Hahn et al. 2005; Trombly et al. 2009; Xu and Gridley 2013; Vanorny et al. 2014). However, the abnormal follicle formation seen in cultured ovaries relates more to the loss of the ability to arrange oocytes by enclosing them with granulosa cells, a basement membrane, and theca cells (in this order), rather than to MOFs. This resembles the phenotype of *Foxl2*-null mice (Uda et al. 2004). Although follicle structure can somehow be successfully created by inhibiting estrogen receptors, the expression of *Foxl2* has been revealed to be high in cultured ovaries, independent of ICI addition (Table 1) (Morohaku et al. 2016b; Tanimoto et al. 2021). In addition, there are almost no primordial follicles in cultured ovaries, and follicles enter the growth stage almost all at once. Leucine-rich repeat containing G-protein-coupled receptor 5 (LGR5)-positive pre-granulosa cells eventually become FOXL2-positive granulosa cells, but reports have suggested that primordial follicles consisting of LGR5-positive pre-granulosa cells are primordial follicles that grow in the second wave or later (Rastetter et al. 2014). It is thus necessary to analyze whether the spatiotemporal expression patterns of FOXL2 and LGR5 are controlled in cultured ovaries, as well as in in vivo studies. Recently, the causes of skipping the primordial follicle state in vitro have been shown to be related to the oxygen concentration and tension generated by the extracellular matrix (Nagamatsu et al. 2019; Shimamoto et al. 2019; Nagamatsu 2021). Furthermore, theca cell layers are extremely thin in cultured ovaries, independent of ICI addition (Fig. 4). This is another issue that needs to be resolved. Abnormalities that occur in in vitro follicle formation are still poorly understood but identifying their causes will improve current understanding of the mechanisms of normal in vivo follicle formation.

## Data Availability

Transcriptome datasets were obtained from DRA010141 archived in the DNA Data Bank of Japan Sequence Read Archive.
